# Determinants of RNA Quality from FFPE Samples

**DOI:** 10.1371/journal.pone.0001261

**Published:** 2007-12-05

**Authors:** Silke von Ahlfen, Andreas Missel, Klaus Bendrat, Martin Schlumpberger

**Affiliations:** 1 QIAGEN GmbH, Hilden, Germany; 2 Gemeinschaftspraxis für Pathologie Niendorf und Hamper, Hamburg, Germany; The Babraham Institute, United Kingdom

## Abstract

The large archives of formalin-fixed paraffin-embedded (FFPE) tissue specimens that exist are a highly valuable source of sample material for molecular biological analysis, including gene expression profiling. However, current data on adverse effects of standard pathological practice on the usefulness of biomolecular analytes obtained from such archived specimens is largely anecdotal. Here, we present a systematic examination of the most relevant parameters for integrity and useability of RNA obtained from FFPE samples, including storage time and conditions, fixation time, and specimen size. The results are particularly relevant for any application relying on cDNA synthesis as an initial step of the procedure, such as RT-PCR, and microarray analysis.

## Introduction

For preservation of tissue samples, formalin fixation followed by embedding in paraffin has been the method of choice for decades, mostly because this treatment maintains morphological features of the original tissue particularly well. With the development of molecular biology techniques for research and, increasingly, diagnostic purposes, there is a growing interest to use the vast archives of formalin-fixed paraffin-embedded (FFPE) tissue samples in these applications as well [1]. The process of fixation and embedding itself [2]–[5] as well as storage of the embedded samples over an extended time period [6] clearly have a decidedly negative impact on the quality of DNA and RNA that can be isolated from such samples. However, relatively little is known about the individual impact of each of these different variables on the usefulness of samples for molecular applications, such as PCR. Such information can be helpful to predict potential usefulness of existing FFPE material for molecular studies, and perhaps also for optimization of sample management for the future.

On a molecular basis, the relevant issues are nucleic acid fragmentation, and modification by chemical reaction between formaldehyde and nucleic acids, including crosslinking with proteins and other biomolecules [2]. While chemical modification and crosslinking by formaldehyde is a direct consequence of the fixation process, fragmentation of RNA can result from a number of different sources: In the time period between sample acquisition and effective fixation, cellular processes and tissue autolysis can result in degradation of RNA, as well as other cellular components [7]. Incubation at elevated temperatures during the embedding process, and prolonged storage of the embedded samples can result in increased fragmentation. Storage of sections can result in oxidation of RNA on the exposed surface, and staining procedures performed on sections may adversely affect RNA quality as well.

In this study, we discern the influences that fixation and embedding, storage time and conditions have on the integrity of RNA isolated from FFPE samples, and demonstrate how these parameters affect RNA performance in cDNA synthesis and RT-PCR applications.

## Results

### Storage of FFPE Specimens

To assess the effects of storage time and conditions on the quality of RNA from FFPE samples, freshly prepared paraffin blocks containing formalin-fixed specimens from different rat tissues (liver, kidney, heart, brain, lung and spleen) were stored at different temperatures (room temperature (20–25°C), 4°C and 37°C). Blocks were also stored at room temperature either open, or protected from air (under nitrogen), and/or sunlight. From each sample, 10 µm sections were cut for subsequent RNA isolation after 0, 1, 3, 6, and 12 months storage time, and RNA was purified at each time point from 1–4 sections using the RNeasy FFPE kit. The RNA obtained was quantified by OD_260_ measurement and tested for the extent of RNA fragmentation using capillary electrophoresis (2100 BioAnalyser, Agilent).

**Figure 1:**
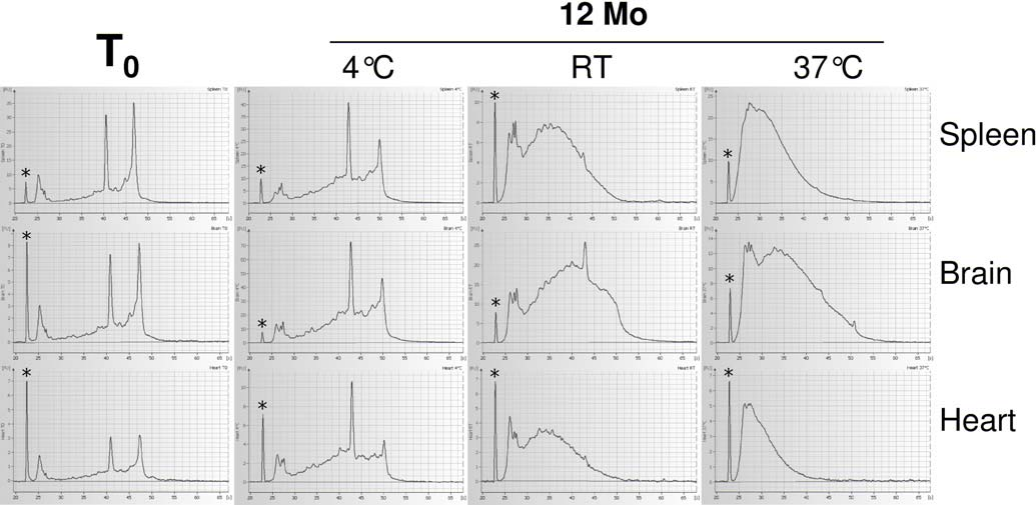
Integrity of RNA isolated from fresh and aged FFPE samples. Rat tissue samples were formalin-fixed and embedded in paraffin. 10 µm sections were cut within 3 days after embedding (T0), or after 1 year storage at the indicated temperatures (RT–room temperature, 20–25°C). Integrity of RNA isolated from individual sections was analysed by capillary electrophoresis. 18S and 28S rRNA bands correspond to 41–43 and 47–50 [s], respectively. The lower marker (marked ‘*’) corresponds to fluorescence intensity of 7–10 [FU].

Fig. 1 shows that storage at different temperatures has a profound influence on the extent of RNA fragmentation. RNA isolated from FFPE specimens within 1–3 days after embedding was surprisingly intact, with RNA Integrity Numbers (RIN [8]) around 7 or higher. Even after 1 year storage at 4°C, ribosomal RNA bands were still clearly visible in most RNA eluates, and RIN values around 5–6 could be obtained. In contrast, RNA from blocks stored at room temperature (20–25°C) or 37°C, did not show clearly distinct rRNA bands anymore, and the mean fragment length was well below 2000 and 100 nucleotides, respectively. Samples derived from different organs did not show any significant differences in the extent of fragmentation over time. Sampling the same FFPE block repeatedly over the course of 12 months showed that fragmentation proceeds gradually over time (data not shown).

**Figure 2:**
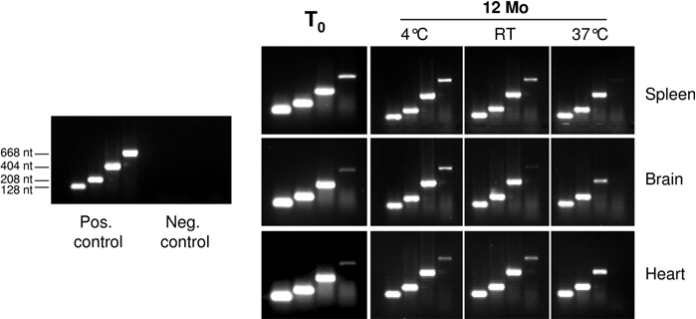
RT-PCR efficiency with RNA from fresh and aged FFPE samples. Rat tissue samples were formalin-fixed and embedded in paraffin. 10 µm sections were cut within 3 days after embedding (T0), or after 1 year storage at the indicated temperatures (RT–room temperature, 20–25°C). The isolated RNA was used for one-step RT-PCR with different primer pairs directed against the rat HPRT gene. Amplicon sizes were 128, 208, 404, and 668 nt. All amplicons were amplified with equal efficiency from RNAlater-stabilized rat tissue (pos. Control), and no products were obtained in no template controls (neg. control).

To determine in how far storage time and conditions affect usefulness of RNA isolated from FFPE material, one-step RT-PCR amplification was performed with primer sets directed against the rat HPRT gene, producing amplicons of different lengths from about 100 to 1100 nucleotides. Notably, even with RNA isolated from FFPE sections within three days after embedding, amplification of fragments up to 700 nt was successful (Fig. 2), whereas longer amplicons (1100 nt) could not be obtained. All amplicons were produced with similar efficiency using RNA isolated from RNAlater-stabilized tissue (not shown). Since BioAnalyser data (Fig. 1) clearly show that RNA up to 4.8 kb in length (size of the 23S rRNA band) can easily be isolated from these FFPE samples, and assuming that the same is true for mRNA, the limiting factor in this case is not RNA length per se. Instead, cDNA synthesis from the isolated RNA is apparently limited by the extent of chemical modification of RNA by formaldehyde during tissue fixation. The RNA purification method used in this study includes a heating step specifically designed to reverse formaldehyde crosslinking, which improves C_T_ values in real-time RT-PCR by as much as 5–6 cycles compared to samples isolated without this step (data not shown). However, previous work has shown that a significant part of the crosslinks formed by reaction of formaldehyde with amino groups in nucleic acid bases and proteins are irreversible [2]. These irreversible chemical modifications of RNA are apparently the limiting factor for RT-PCR in samples isolated shortly after fixation and embedding. Samples stored for up to 12 months at room temperature or 4°C did not show a significant decline in performance, despite the increasingly fragmented state of the isolated RNA. In contrast, samples stored for 12 months at 37°C yielded RNA material which did not allow amplification of fragments longer than 400 nt, indicating that in this case fragmentation had become the limiting factor.

Exposure of individual FFPE sections to light and air had a negative influence on RNA quality, but storage of entire FFPE blocks either in the open or protected from air and light did not influence the effect of storage time on RNA quality, as long as the uppermost section was discarded at each time point, and sections not directly exposed to air were used for RNA preparation (data not shown).

In real-time PCR applications, amplicons are usually shorter than 200 nt. However, in cases where oligo-dT priming is used for cDNA synthesis, the distance between the 3′ end of the mRNA and the 5′ end of the PCR forward primer becomes the limiting factor, because shorter cDNA products may not contain the entire region to be amplified. Consequently, RT-PCR amplicons should be within ∼500 nt or less from the mRNA 3′ end when oligo-dT priming is used for cDNA synthesis. This is not a concern when random or gene-specific primers are used for cDNA synthesis. Similar considerations regarding cDNA length and distance from the 3′ prime end apply to other workflows that rely on cDNA synthesis, such as microarray hybridizations, in cases where oligo-dT priming is used.

### Influence of Fixation Time

Another critical factor for nucleic acid quality from FFPE samples are the conditions used for formalin fixation. It is crucial to keep the time between sample acquisition and fixation as short as possible in order to avoid tissue autolysis and nucleic acid degradation by endogenous nucleases. In addition, overfixation can become an issue, particularly if fixation proceeds for considerably longer than 24 hours, resulting in more irreversible crosslinks [3]–[5]. There are also reports in the literature that fixation by formaldehyde, particularly over extended time periods, results in increased RNA degradation [9].

**Figure 3:**
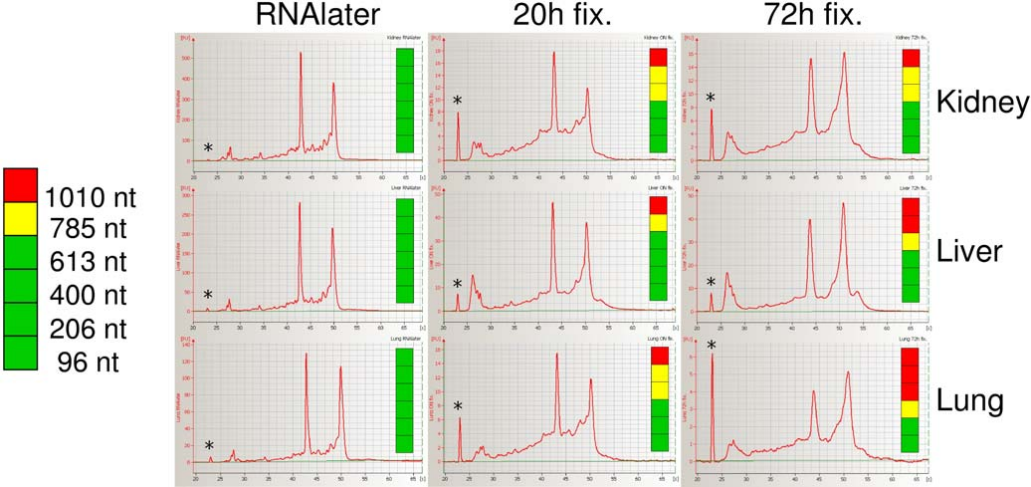
Effect of fixation on RNA integrity and RT-PCR performance. Rat tissue samples were either stabilized in RNAlater or formalin-fixed, either over night or for 3 days. Fixed samples were embedded in paraffin, and 10 µm sections were cut within 3 days after embedding for preparation of RNA. RNA quality from each sample type was analysed by capillary electrophoresis. 18S and 28S rRNA bands correspond to 42–44 and 49–51 [s], respectively. The lower marker (marked ‘*’) corresponds to fluorescence intensity of 6–8 [FU]. The color bars indicate RNA performance in one-step RT-PCR using different primer pairs directed against the rat RPL4 gene. Amplicon sizes from top to bottom were 1010, 785, 613, 400, 206, and 96 nt. Red indicates no amplification, yellow–weak amplification, green–good amplification.

To assess the influence of overfixation on RNA quality, we compared tissue samples fixed either overnight or for 72 hours in formalin, compared to RNAlater-stabilised tissue samples from the same animal. BioAnalyser data show that even after 72 h fixation in formalin, the RNA is only marginally fragmented, and ribosomal RNA bands are still largely intact (Fig. 3), thus clearly showing that the reaction with formaldehyde itself does not cause nucleic acid fragmentation. In contrast, RT-PCR results demonstrate that prolonged fixation aggravates the negative effects of formaldehyde modification on cDNA synthesis. Whereas amplicons up to 800 nt can be routinely amplified from samples fixed over night, the maximum amplicon length for samples fixed for 72 hours in our PCR system was around 400–600 nt in most cases (Fig. 3).

While overfixation did not directly result in increased RNA fragmentation, our preliminary data indicate that overfixation may result in faster RNA fragmentation during storage of FFPE blocks (data not shown).

### Influence of Specimen Size

With the exception of small biopsies, penetration of the tissue by the fixative is the rate-limiting step in fixation, with initial penetration rates for formalin around 1 mm per hour. Penetration rates decrease with depth. Thus, around 8 hours are required for penetration of 5 mm tissue [10]. Therefore, it is generally recommended to keep sample thickness around or below 5 mm for efficient and even fixation, in order to avoid the risk of overfixation at the periphery, and tissue autolysis near the center of the specimen [7], [11].

**Figure 4:**
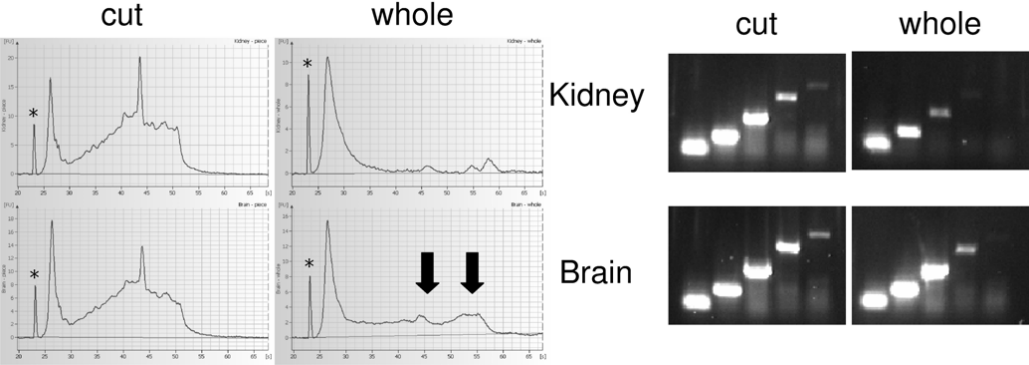
Effect of sample size during fixation on RNA integrity and RT-PCR performance. Rat tissue samples were formalin-fixed over night, either as whole organs, or cut to pieces <3 mm thickness. Samples were embedded in paraffin, and 10 µm sections were cut within 3 days after embedding. RNA quality was analysed by capillary electrophoresis. The lower marker (marked ‘*’) corresponds to fluorescence intensity of 8–9 [FU]. RNA performance was tested in one-step RT-PCR using different primer pairs directed against the rat RPL4 gene. Amplicon sizes as seen on the gel images were 96, 206, 400, 613, and 785 nt.

We examined the effect of specimen size and thickness by comparing rat kidney and brain samples that were either fixed whole (about 1 cm thick), or cut into smaller pieces, around 3–4 mm thick (Fig. 4). Analysis of RNA purified from these FFPE samples within days after embedding showed strong RNA fragmentation when entire organs where fixed, compared to relatively intact RNA obtained from thinner pieces. The observed fragmentation also resulted in shorter maximum amplicon size in our RT-PCR system. In the electropherograms of RNA from the large specimens, a small amount of longer RNA is still visible (Fig. 4, arrows). Presumably the respective RNA originates from the outer parts of the specimens which were penetrated by the fixative more quickly. The larger amount of these longer RNA molecules in the brain sample is also reflected in longer maximal amplicon size of 600 nt, compared to 400 nt in kidney (Fig. 4).

The degradation effect observed here is surprisingly strong, considering that the thickness of the intact rat organs used was around 1 cm only. It is possible that the intact organs are more slowly penetrated compared to equally sized pieces cut from larger tissues.

### RNA from Pathology Samples

**Figure 5:**
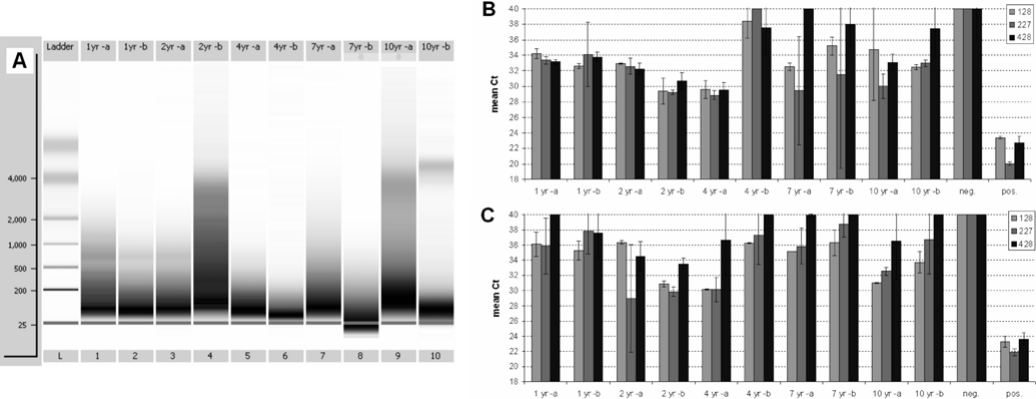
Fragmentation state and RT-PCR performance of RNA from tumor samples. RNA was isolated from individual 10 µm sections of FFPE tumor samples of different age (1–10 years). Fragmentation state was analysed by capillary electrophoresis (A). RNA performance was tested in 2-step real-time RT-PCR using assays directed against the human TBP gene, using either a mix of random and oligo-dT primers for cDNA synthesis (B), or oligo-dT alone (C).

To compare our results from FFPE specimens obtained from animal tissues under strictly controlled conditions with real-life pathology samples, FFPE specimens from surgically removed lung carcinoma were obtained (see Methods). The FFPE samples had been stored for 1, 2, 4, 7, and 10 years at ambient temperature prior to sectioning. RNA preparation was done using the same purification protocol as for the rat samples above. RNA yields from a single 10 µm section were between 2.5 and 16 µg total RNA, with no correlation between age of sample and RNA yield. RNA integrity was checked by capillary electrophoresis (Fig. 5a). According to the electropherograms, mean size of RNA fragments isolated was between 220 and less than 100 nt, also with no correlation between age of sample and mean fragment size. Even for the 1 year-old specimens, RNA integrity was significantly inferior to the rat samples after 1 year storage at 37°C (Fig. 5a, and Fig. 1). The reason for this is most likely tissue autolysis, because thickness of the cancer specimens used for fixation was generally more than 2 cm, which implies more than 24 hours for penetration of the sample by the formalin [10].

To determine the usefulness of such samples for molecular analysis, real-time RT-PCR was performed on the RNA samples. Different primer pairs directed against the human housekeeping gene TBP mRNA were used to amplify fragments at different distances from the 3′ end (128, 227, and 428 nt). For cDNA synthesis, the QuantiTect RT kit (QIAGEN) was used, either with a primer mix composed of random and oligo-dT primers (as contained in the kit), or, for comparison, with oligo-dT primer. Using the primer mix, all of the 1- and 2-year old samples and one of the 4-year samples produced threshold cycle (C_T_) values of 29–35 (Fig. 5b), whereas the remaining samples gave higher C_T_ values, and in some cases failed to produce any meaningful signals within 40 PCR cycles. Although no clear correlation between RNA fragmentation and C_T_ value was observed, the most fragmented RNA sample (4yr –b, lane 6 in Fig. 5a) gave the poorest PCR results. In contrast, using oligo-dT priming for cDNA synthesis resulted in roughly comparable C_T_ values for the amplicon located at 127 nt from the 3′ end, but significantly higher values for the more distant amplicons (Fig. 5b). Only 2 samples produced meaningful results consistently for the amplicon located 428 nt from the 3′ end. Using a different reverse transcriptase for cDNA synthesis produced similar results (data not shown).

## Discussion

Our data clearly show two distinguishable factors that influence usefulness of RNA isolated from FFPE sections. Under the assumption that rRNA and other RNA species (most notably mRNA) show similar degradation kinetics, integrity of RNA can be assessed using electrophoretic methods, and depends mainly on the quality of the fixed tissue sample (time between resection and effective fixation), storage time and conditions of the paraffin blocks (Fig. 1), and presumably embedding conditions (primarily the incubation time and temperature in hot paraffin). The second factor, chemical modification of RNA by formaldehyde, is not resolved by electrophoretic assays, but has a strong negative effect on RT-PCR, and presumably other enzymatic procedures as well. Because the actual PCR template is newly synthesized cDNA, the observed negative effects in our assays must be due to inhibition of the reverse transcription reaction. The degree to which chemical modification impacts RNA quality depends on fixation time (Fig. 3), and also on the RNA isolation procedure.

The RNA isolation procedure we used involves a short proteinase K digestion, followed by incubation at 70°C under conditions that promote breakage of methylene crosslinks which result from formalin fixation. Proteinase K digestion is necessary to release RNA from the meshwork of crosslinked protein and nucleic acids by digesting the protein portion of the crosslinked molecules, potentially down to the level of tetrapeptides [12]. However, it does not attack the actual methylene bridge that forms the crosslink, because it does not involve a peptide bond. In contrast, the heating step breaks the actual crosslinks, but is limited by the heat tolerance of the RNA itself, and by the irreversible nature of part of the crosslinks [2]. Accordingly, it can be assumed that the inhibitory effect of RNA from FFPE samples on cDNA synthesis that we observed is due mainly to very short peptides that are irreversibly crosslinked to RNA bases.

Even though integrity of RNA isolated from FFPE specimens clearly deteriorates with storage time, performance in RT-PCR assays is relatively poor even for seemingly intact RNA, and changes relatively little during the first 12 months of storage at room temperature–indicating that the limiting factor is chemical modification by formaldehyde. Only after storage for more than 12 months or at elevated temperatures, RNA integrity becomes the limiting factor for RT-PCR performance–roughly in agreement with the fragment length observed in electrophoresis of RNA from such aged samples.

Fixation with formaldehyde efficiently and irreversibly inactivates endogenous RNases. Thus, while RNA integrity is a poor predictor of performance for RNA from relatively fresh FFPE samples, it provides valuable information about RNA damage and tissue autolysis that occurred prior to embedding, i.e. in the time between resection and the onset of fixation. For FFPE specimens stored over a longer time period (months or years), the fragmentation state of the isolated RNA reflects both the storage time and conditions (temperature), and also the treatment of the samples prior to embedding.

The cancer specimens we tested demonstrate that RT-PCR results can be obtained even from FFPE samples that yield strongly degraded RNA, although the required RNA input may be higher than for intact RNA, and protocols may have to be adapted. The lack of correlation between RNA integrity and storage time, even for those FFPE blocks less than 2 years old, indicates that RNase activity was not destroyed quickly after surgical removal of the tumor tissue, presumably because large size of the fixed specimens prevented fast enough penetration of formaldehyde throughout the tissue.

For clinical practice, where FFPE specimens are collected for potential use in gene expression analysis, or any molecular biology assays in addition to histopathology, the following recommendations should be considered where possible: The time between surgical removal of the specimen and fixation in formalin should be as short as possible, and the tissue should not be thicker than approximately 5 mm to allow quick and efficient inactivation of RNases and other enzymatic acitvities that may influence gene expression profiles. RNA should preferrably be isolated within 1 year after embedding, i.e. before fragmentation during storage of FFPE samples becomes limiting for performance of the RNA in enzymatic assays. Finally the RNA isolation procedure should be optimized to reverse effects from formaldehyde crosslinking as much as possible. For cDNA synthesis, oligo-dT priming should be avoided, unless all target sequences to be analysed are within 100–500 nt of the mRNA 3′ end, depending on RNA integrity (Table 2).

**Table 1:** Oligonucleotide Primer Sequences for RT-PCR.

Target rat gene	Amplicon length	Forward Primer	Reverse Primer
HPRT	128 nt	AGACGTTCTAGTCCTGTG	CCTACAGGCTCATAGTGCAAA
HPRT	208 nt	AAAGCCAAGTACAAAGCCTAAA	CCTACAGGCTCATAGTGCAAA
HPRT	404 nt	CAAGAGTCCTGTTGATGTG	GGGAAACCTCTTAGATGCTGT
HPRT	668 nt	TTGCTGACCTGCTGGATT	TCTGTCTGTCTCACAAGGGAA
HPRT	1065 nt	GCGTCGTGATTAGTGATGATG	GGGAAACCTCTTAGATGCTGT
Rpl4	96 nt	GATGAGTTGTATGGCACTTGG	GATTCTGCTAAGGTCTGTGTTC
Rpl4	206 nt	GAAACATCCCTGGTATTACTCT	GCTAAGGTCTGTGTTCATCATC
Rpl4	400 nt	CATCCCTGGTATTACTCTGCTT	GGCTTCCAGCTTTTTCACTC
Rpl4	613 nt	CAGAGAATGAGAGCTGGCAA	CATCCACAGGCTTCTTTCCTTT
Rpl4	822 nt	TCATCTGGCAAGAATGTCACT	GCTAAGGTCTGTGTTCATCATC
Rpl4	1020 nt	TCATCTGGCAAGAATGTCACT	GGCTTCCAGCTTTTTCACTC
Target human gene	Distance from 3′ end	Forward Primer	Reverse Primer
TBP	128 nt	TGCCAGACACATTCCACCTCTC	CAGCCATTCATTTTCATTAACACATA
TBP	227 nt	TTCCCCATGAACCACAGTTTTT	CTGCAATACTGGAGAGGTGGAATGT
TBP	428 nt	GGGAAGGGGCATTATTTG	CCAGATAGCAGCACGGTA

**Table 2:** Considerations for sample handling to improve RNA yields and quality from FFPE samples.

Problem	Recommendation
Tissue autolysis	Start fixation as soon as possible
Tissue autolysis	Cut samples into thin pieces (preferrably 5 mm or thinner)
Efficiency of cDNA synthesis	Avoid overfixation
Efficiency of cDNA synthesis	RNA isolation should include crosslink reversal step
RNA integrity	Isolate RNA preferrably within 1 year after fixation and embedding
RT-PCR performance	Use of random priming or primer mix instead of oligo-dT

Although our analysis was limited to RT-PCR applications, the same considerations should apply to microarray analysis and other applications that rely on cDNA synthesis as well.

## Materials and Methods

### Tissue samples

Organs were prepared from adult Wistar rats, cut to appr. 3–5 mm thick pieces, and immediately submerged in 10% neutral buffered formalin for fixation and embedding in paraffin, or stabilized in RNAlater solution (QIAGEN GmbH, Hilden, Germany), for reference samples with ideal RNA preservation, respectively. Formalin fixation of tissue specimens was performed over night (20–24 h) at room temperature, unless otherwise noted. Specimens were dehydrated in 70%, 90%, and 100% ethanol, followed by xylene, for 2×1 h each solution, then embedded in Paraplast Plus (Sherwood Medical Co., St. Louis) for 2–3 h at 60°C. For RNA isolation, 10 µm sections were cut on a standard microtome. In each case, the first 2 sections were discarded to exclude negative effects from exposure to air.

FFPE specimens from surgically removed lung carcinoma were obtained using routine diagnostic procedures; the study was approved by the ethical committee of the Ärztekammer Hamburg.

### RNA preparation

For preparation of RNA from FFPE samples, the RNeasy FFPE kit (QIAGEN GmbH, Hilden, Germany) was used according to manufacturers recommendations. Depending on the tissue, 1 to 4 sections of 10 µm thickness were used per preparation. For RNA preparation from RNAlater stabilized samples, RNeasy plus or RNeasy Lipid Tissue kits were used. RNA yields were determined by A_260_ measurement, RNA integrity was assessed by microcapillary electrophoresis (2100 BioAnalyser, Agilent Technolologies, Waldbronn, Germany).

### RT-PCR assays

Primer pairs were designed to generate amplicons of different lengths from the rat HPRT (128, 208, 404, 668, and 1065 nt) and Rpl4 (96, 206, 400, 613, 785, and 1010 nt) mRNAs, respectively (NM_012583, NM_022510). See table 1 for primer sequences. One-step RT-PCR was performed using the QIAGEN OneStep RT-PCR Kit (QIAGEN GmbH, Hilden, Germany) according to manufacturer's recommendations (thermal cycling conditions: 45°C for 60 minutes (RT step) and 94°C for 15 minutes, followed by 35 cycles of 94°C for 30 seconds, 55°C for 30 seconds, and 72°C for 1 minutes, and a final extension step for 10 minutes at 72°C), and the RT-PCR products were analysed by agarose gel electrophoresis.

For human samples, real-time RT-PCR assays were designed for the TBP gene, with the amplicons at different distances from the mRNA 3′ end (128, 227 and 428 nt, counted from the 5′ end–see table 1 for primer sequences) but comparable lengths. Two-step RT-PCR was performed using the QuantiTect Reverse Transcription Kit (QIAGEN GmbH, Hilden, Germany) for cDNA synthesis either according to manufacturer's recommendations, or using oligo-dT primer in place of the supplied primer mix, followed by PCR using the QuantiTect SYBR Green PCR Kit (QIAGEN GmbH, Hilden, Germany). Thermal cycling conditions following the 15 minutes at 94°C activation step were: 15 seconds at 94°C, 30 seconds at 50°C, 30 seconds at 72°C for 40 cycles.
